# Continued Colonization of the Human Genome by Mitochondrial DNA

**DOI:** 10.1371/journal.pbio.0020273

**Published:** 2004-09-07

**Authors:** Miria Ricchetti, Fredj Tekaia, Bernard Dujon

**Affiliations:** **1**Unité de Génétique Moléculaire des Levures (UFR 927 Univ. P. et M. Curie and URA 2171 CNRS), Department of Structure and Dynamics of GenomesInstitut Pasteur, Paris, France; **2**Unité de Génétique et Biochimie du Développement (URA 1960 CNRS), Department of ImmunologyInstitut Pasteur, ParisFrance

## Abstract

Integration of mitochondrial DNA fragments into nuclear chromosomes (giving rise to nuclear DNA sequences of mitochondrial origin, or NUMTs) is an ongoing process that shapes nuclear genomes. In yeast this process depends on double-strand-break repair. Since NUMTs lack amplification and specific integration mechanisms, they represent the prototype of exogenous insertions in the nucleus. From sequence analysis of the genome of *Homo sapiens,* followed by sampling humans from different ethnic backgrounds, and chimpanzees, we have identified 27 NUMTs that are specific to humans and must have colonized human chromosomes in the last 4–6 million years. Thus, we measured the fixation rate of NUMTs in the human genome. Six such NUMTs show insertion polymorphism and provide a useful set of DNA markers for human population genetics. We also found that during recent human evolution, Chromosomes 18 and Y have been more susceptible to colonization by NUMTs. Surprisingly, 23 out of 27 human-specific NUMTs are inserted in known or predicted genes, mainly in introns. Some individuals carry a NUMT insertion in a tumor-suppressor gene and in a putative angiogenesis inhibitor. Therefore in humans, but not in yeast, NUMT integrations preferentially target coding or regulatory sequences. This is indeed the case for novel insertions associated with human diseases and those driven by environmental insults. We thus propose a mutagenic phenomenon that may be responsible for a variety of genetic diseases in humans and suggest that genetic or environmental factors that increase the frequency of chromosome breaks provide the impetus for the continued colonization of the human genome by mitochondrial DNA.

## Abstract 2

DNA from mitochondria has regularly inserted into the human nuclear genome. Some insertions are polymorphic, revealing that the invasion of the human genome is an ongoing process

## Introduction

Insertion of new sequences into nuclear DNA has a major impact on its architecture and is an important mechanism for the evolution of eukaryotic genomes. Moreover, when targeted to gene loci, these insertions can be mutagenic, and in humans this process contributes to a number of diseases (Deininger and Batzer 1999; Neil and Cameron 2002; Nelson et al. 2003). The frequency of the insertion events and the site of integration are therefore critical factors influencing genomic stability. In humans these two aspects have been investigated for mobile elements, including long and short interspersed elements and retroviruses (Li et al. 2001; Batzer and Deininger 2002), but much less is known about nuclear DNA sequences of mitochondrial origin (NUMTs), which have been found associated with diseases in humans (Willett-Brozick et al. 2001; Borensztajn et al. 2002; Turner et al. 2003).

DNA fragments of mitochondrial origin, originating from both coding and noncoding regions, are found as sequence fossils in the nuclear genomes of various eukaryotes (Blanchard and Schmidt 1996). However, de novo integrations have been recently detected in yeast and humans (Ricchetti et al. 1999; Yu and Gabriel 1999; Turner et al. 2003), and insertion of NUMTs in the nuclear genome has been found to be an ongoing process in yeast (Ricchetti et al. 1999). We and others have previously shown that NUMTs integrate in the nuclear genome during the repair of double-strand breaks (DSBs) in yeast growing mitotically (Ricchetti et al. 1999; Yu and Gabriel 1999). In these studies, sequences of mitochondrial origin were the main or the exclusive type of DNA able to integrate at an induced DSB.

Before the sequencing of the human genome was completed, occasional reports described sequences of mitochondrial origin located in the nucleus (Tsuzuki et al. 1983; Perna et al. 1996), in one case in vivo (Zischler et al. 1995). More recently, sequence analysis performed on the first human genome draft revealed the presence of between 280 and 296 NUMTs (Mourier et al. 2001; Tourmen et al. 2002). According to one study, it appears that only one third of NUMTs were integrated as new sequences, whereas the remaining two thirds originated as duplications of preexisting NUMTs (Hazkani-Covo et al. 2003). Another report suggests that most NUMTs arose from independent insertion events (Bensasson et al. 2003), thereby raising questions regarding the real insertion rate of these sequences in the human genome. Moreover, it has been suggested that most NUMTs have been inserted in a primate ancestor (Tourmen et al. 2002; Bensasson et al. 2003). Thus, the rate and the effects of colonization of the human genome by DNA fragments of mitochondrial origin remain unclear, and the presence of such sequences has not been fully investigated in humans. With the availability of the human genome sequence coupled with significant discoveries on the evolution of *Homo sapiens,* experimental approaches that compare individuals within this species and its closest relative, the chimpanzee, can be undertaken (Chen et al. 2001).

In the present study, we demonstrate the presence in vivo of NUMTs in the human and in the chimpanzee genomes, using genome-wide sequence analysis combined with direct evaluation of DNA samples of individuals. Moreover, we show a significant degree of insertion polymorphism of NUMTs in human populations. We also provide a comprehensive analysis of NUMTs that have specifically colonized the human genome, and we determine the fixation rate of these sequences in *H. sapiens.* Furthermore, we correlate these findings with observations of the insertion of NUMTs involved in human diseases. We show that human-specific NUMTs*,* unlike those in yeast, preferentially integrate in known or predicted genes and can therefore be mutagenic, thereby generating genetic alterations in humans.

## Results

### NUMTs Are Mitochondrial Sequences Residing in the Human Nuclear Genome

From a blastn search on the database of H. sapiens published by the public consortium (Lander et al. 2001), using as query the human mitochondrial (mt) DNA sequence (Anderson et al. 1981), 211 NUMTs were found. We observed that approximately 93% of NUMTs represent insertions of a single DNA fragment, whereas 7% consist of multiple, unrelated mtDNA fragments, similar to the pattern frequently found in yeast (Ricchetti et al. 1999). We observed that NUMTs ranged in size from 47 to 14,654 bp with a sequence identity to the human mtDNA of 78%–100%. Previous analyses were based on less complete genome sequencing (84% complete for the most recent study; Bensasson et al. 2003), while our study was performed on a 99% complete sequencing of the euchromatic genome of *H. sapiens.*

Our updated analysis (data not shown) reveals that the majority of these NUMTs correspond to those previously documented (Mourier et al. 2001; Tourmen et al. 2002). However, our study draws attention to NUMTs that are highly identical to mtDNA, and some of these sequences did not appear in earlier analyses. Indeed most of the NUMTs in our study are shorter than 100 bp (see Table 1), whereas former investigations focused more on longer NUMTs. Although the presence of shorter NUMTs has been reported, these have not been published (Tourmen et al. 2002). Combining the lowered size threshold and the more complete database, we were able to identify 23 new NUMTs (labeled with an asterisk in Table 1). Most of the new sequences are of the highest interest for our studies of the acquisition of NUMTs by H. sapiens and of insertion polymorphism in humans (see below).

**Table 1:**
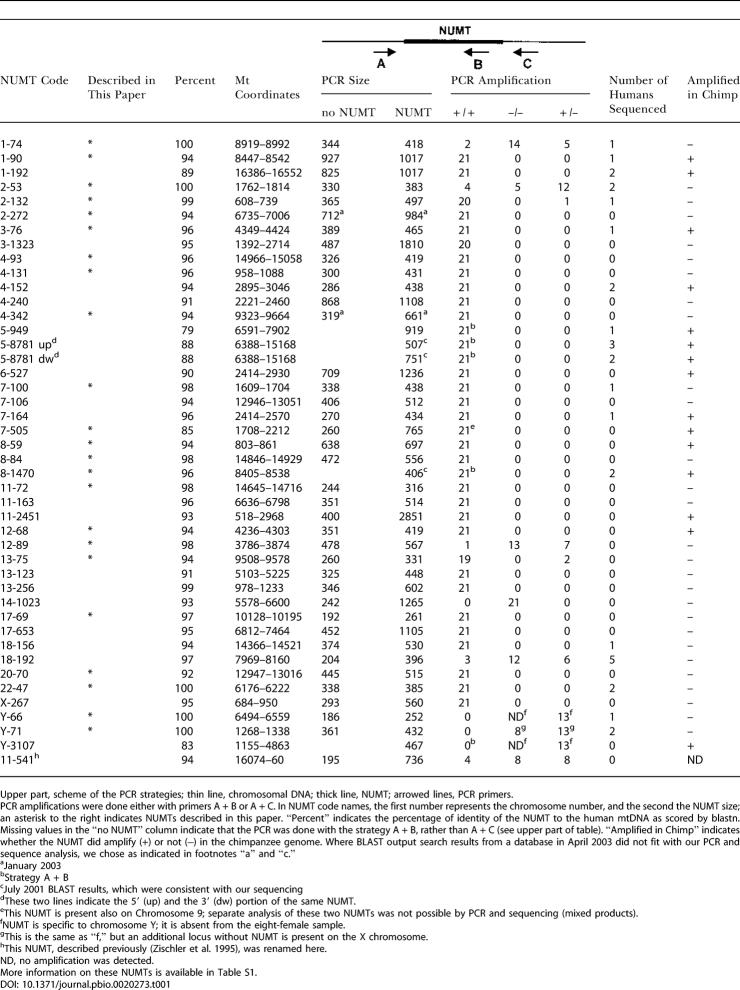
PCR Amplification and Sequence Analysis of NUMTs from Humans and Chimpanzees

Upper part, scheme of the PCR strategies; thin line, chromosomal DNA; thick line, NUMT; arrowed lines, PCR primers

PCR amplifications were done either with primers A + B or A + C. In NUMT code names, the first number represents the chromosome number, and the second the NUMT size; an asterisk to the right indicates NUMTs described in this paper. “Percent” indicates the percentage of identity of the NUMT to the human mtDNA as scored by blastn. Missing values in the “no NUMT” column indicate that the PCR was done with the strategy A + B, rather than A + C (see upper part of table). “Amplified in Chimp” indicates whether the NUMT did amplify (+) or not (−) in the chimpanzee genome. Where BLAST output search results from a database in April 2003 did not fit with our PCR and sequence analysis, we chose as indicated in footnotes “a” and “c.”

^a^January 2003

^b^Strategy A + B

^c^July 2001 BLAST results, which were consistent with our sequencing

^d^These two lines indicate the 5′ (up) and the 3′ (dw) portion of the same NUMT

^e^This NUMT is present also on Chromosome 9; separate analysis of these two NUMTs was not possible by PCR and sequencing (mixed products)

^f^NUMT is specific to chromosome Y; it is absent from the eight-female sample

^g^This is the same as “f,” but an additional locus without NUMT is present on the X chromosome

^h^This NUMT, described previously (Zischler et al. 1995), was renamed here

ND, no amplification was detected

More information on these NUMTs is available in Table S1

To determine whether sequences of mitochondrial origin were actually integrated in the human nuclear genome, and were not a result of contamination of DNA library preparations, we selected 42 NUMTs for analysis in human samples. Our choice included the 36 NUMTs with the highest identity (91% to 100%) to the mtDNA, one NUMT having the longest stretch of DNA with high identity (88%), one NUMT corresponding to the highly variable region of the mtDNA (D-loop) (Cann and Wilson 1983), and four NUMTs randomly chosen with identity from 79% to 90% (Table 1). Each of these NUMTs was amplified by PCR from DNA obtained from 21 human donors (eight females, 13 males) representing different ethnical groups. Our pool consisted of ten Caucasians, seven Africans (including four Pygmies), two Japanese, and two Chinese (Table 2). To amplify chromosomal NUMTs and avoid amplification of the mt chromosome, we used a primer located in the upstream flanking region, in combination with a primer located either in the 3′ region of the NUMT or in the downstream flanking region (see upper part of Table 1, primers A + B or A + C, respectively). In the former case, PCR amplification served as a supplementary control for bona fide mtDNA integration at the locus, while in the latter, PCR amplification was followed by sequencing to assay for the presence of the NUMT. Forty-one out of 42 loci tested amplified a fragment of the expected length (Table 1), while one locus (14–1023 [Chromosome 14; size = 1023 bp]) amplified a fragment not containing the NUMT in all individuals tested. Eighteen loci were analyzed further in one or more individuals by sequencing the amplified fragment to verify whether these DNA fragments included the sequence of mt origin (see Table 1). The expected sequence was indeed present in all cases tested (except at polymorphic loci, described later, and at insertions in the Y chromosome, present only in males).

**Table 2:**
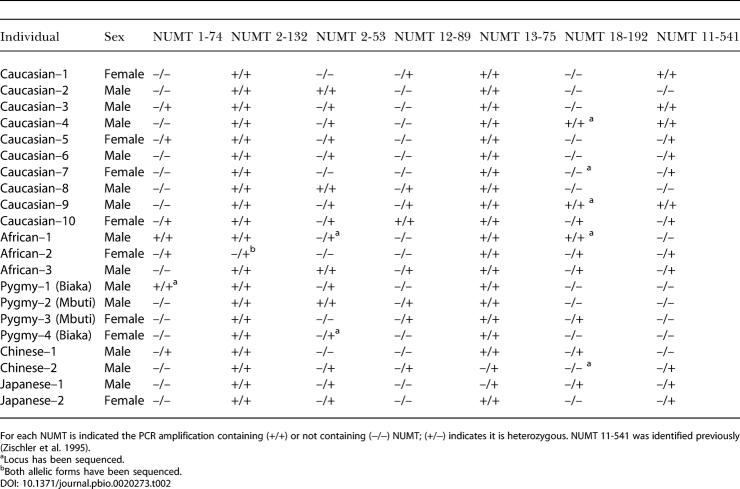
Insertion Polymorphism of NUMTs Displaying Distinct Lineage Characteristics in Humans

For each NUMT is indicated the PCR amplification containing (+/+) or not containing (−/−) NUMT; (+/−) indicates it is heterozygous. NUMT 11-541 was identified previously (Zischler et al. 1995)

^a^Locus has been sequenced

^b^Both allelic forms have been sequenced

In summary, results from two amplification strategies and from sequencing demonstrated that these NUMTs were indeed present at the expected chromosomal location and that they are bona fide mt sequences residing in the human nuclear genome.

### Insertion Polymorphism of NUMTs in Human Populations

**Figure 1:**
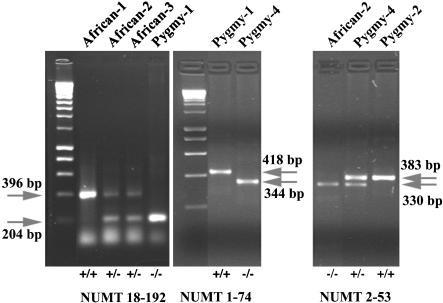
Polymorphism of NUMTs 18-192, 1-74, and 2-53 The polymorphism of NUMTs 18-192, 1-74, and 2-53 as revealed by PCR amplification and electrophoresis of the products on 2% agarose gels. For each locus, the upper arrow indicates the fragment that contains the NUMT, and the lower arrow indicates the fragment that does not contain the NUMT. The individual tested is indicated above. The (+/+) are homozygous positive, (+/−) are heterozygotes, and (−/−) are homozygous negative.

**Figure 2:**
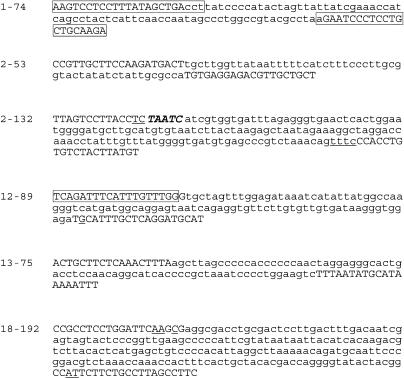
Sequence Insertion Polymorphism of Six NUMTs Sequence of NUMTs 1-74, 2-53, 2-132, 12-89,13-75 and 18-192 are indicated in lower case and the flanking sequences in capital letters. Underlined letters represent nucleotides homologous to both the mt and the chromosomal sequences (microhomology). Bold and italicized letters correspond to nucleotide additions, following the NUMTs insertion, which are absent from the −/− individuals. The individuals sequenced are indicated in Table 2. In all cases the sequence corresponded to the one available on the human genome public Web sites. Boxes represent exon sequences. In 12-89, the exon sequence would extend till the stop codon (taa).

The colonization of human populations by various NUMTs revealed striking disparities. Thirty-five NUMTs were present in homozygous form in all individuals tested (see Table 1). Interestingly, NUMTs 1-74, 2-53, 12-89, and 18-192 were present in only a few individuals either as homozygous or heterozygous loci (see Figure 1; Tables 1 and 2). A more limited heterogeneity was observed for NUMTs 13-75 and 2-132, where only two and one individual, respectively, were heterozygous. In total, six out of 41 NUMTs showed insertion polymorphism. Integration of NUMTs was further confirmed by sequencing both positive and negative samples (see Table 2 for the samples tested). The sequences of these six NUMTs are shown in Figure 2. NUMT 11-541, whose insertion polymorphism was previously described (Zischler et al. 1995; Thomas et al. 1996), was reanalyzed here (Tables 1 and 2). By comparing the flanking sequences of individuals carrying a NUMT with those of individuals who have no NUMT, it is possible to identify the junction sites and to also clarify the mechanism by which NUMTs were inserted. This analysis was done for NUMTs 2-132 and 18-192, in which the junction sites (Figure 2) show microhomology between the invading NUMT and the chromosomal end, and sometimes addition of a few nucleotides. Both the presence of microhomology and the addition of short sequences also accompanied the insertion of NUMTs in the yeast genome (Ricchetti et al. 1999), and they are hallmarks of the DSB repair mechanism non-homologous end-joining (NHEJ) (Critchlow and Jackson 1998). This suggests that NHEJ may also account for the insertion of NUMTs in humans.

Interestingly, one or more of these six NUMTs were detected among individuals within each ethnic group, indicating that their insertion in the nuclear genome occurred soon after the origin of modern humans and that they represent the most recent integrations of our studied cases. Despite the limited sampling size (42 alleles), the frequency of alleles carrying the insertion varies greatly according to the NUMT (calculated from Table 2): 98%, 95%, 48%, 29%, and 21% for NUMTs 2-132, 13-75, 2-53, 18-192, and both 12-89 and 1-74, respectively. Moreover, allele frequencies among different ethnic groups are not equal. For example, NUMTs 1-74 and 18-192 are poorly represented among Caucasians and Asians and are more frequent in non-pygmy Africans. NUMT 12-89, unlike other NUMTs, is poorly represented in non-pygmy Africans. As a result, each NUMT presents a unique population fingerprint.

### Acquisition of NUMTs by H. sapiens

**Figure 3:**
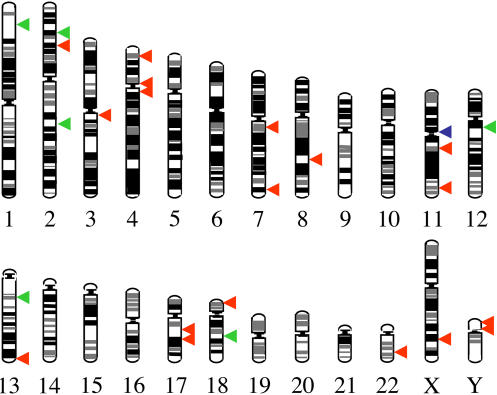
Distribution of Human-Specific NUMTs in Chromosomes A scale representation of the human chromosomes. The location of human-specific NUMTs is indicated with a red arrow. A green arrow indicates the position of NUMTs showing insertion polymorphism in humans, and a blue arrow indicates a previously described NUMT (11-541).

To evaluate which NUMTs are specific to humans, we amplified by PCR the 42 loci described above on chimpanzee DNA *(Pan troglodytes).* For each locus, one to three chimpanzee individuals were analyzed. Forty-two out of 42 primer pairs successfully amplified the target site also in chimpanzees because of the high sequence identity of the two genomes (average 98.7%) (Chen et al. 2001). Only the regions flanking the previously described NUMT 11-541, which is considered separately in our investigation, did not amplify in chimpanzees. Locus 14-1023, where no NUMT was identified in humans, also showed no insertion in the chimpanzee. Surprisingly, only 14 loci contained the NUMT (see Table 1). All of these NUMTs were also found in all human individuals tested, indicating that they were present in the common ancestor of human and chimpanzee. On the contrary, 27 NUMTs absent from the chimpanzee genome represent recent acquisitions in *H. sapiens.* The distribution of these NUMTs in the human chromosomes is shown in Figure 3. All NUMTs whose presence was not found in all human individuals fell in this category. From our data, 24 out of 27 NUMTs specific to humans had greater than 94% of sequence identity to the human mtDNA, and three out of 27 NUMTs had sequence identity of 91%–92%. This higher level of identity is consistent with the idea that NUMTs specific to humans are recent insertions (for the calculation of the insertion time of NUMTs, see Materials and Methods). Similar values of identity to the mtDNA were also found for the recent insertions of mt sequences in the yeast genome (Ricchetti et al. 1999). Seven out of 14 NUMTs present both in humans and in chimpanzees have lower levels of identity to the mtDNA (between 79% and 90%), as expected; however, the remaining seven NUMTs have 94%–96% identity to the mtDNA (see Table 1), indicating that the level of identity per se is not a rigorous criterion for human specificity. Interestingly, most of NUMTs present only in humans are short sequences, and about half of them are less than or equal to 100 bp. In summary, out of 211 NUMTs recognizable in the human genome, at least 27 (or 28, if we also include NUMT 11-541) were specific to humans, and we do not expect this value to increase significantly because 99% of the euchromatic genome of H. sapiens was analyzed, and we assume that most NUMTs with low identity to the mtDNA (less than or equal to 90%) are unlikely to be human-specific. This results in an average of one NUMT integration in the germline for each 180,000 y, in the last 4–6 million years (Myr). Interestingly, one fourth of these NUMTs (6/27, or 7/28 if we include NUMT 11-541) show insertion polymorphism (see Table 1 and above), indicating that they have occurred in more recent times.

### High Frequency of Human-Specific NUMTs in Chromosomes 18 and Y

**Figure 4:**
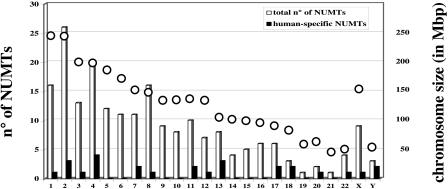
Human-Specific NUMTs in Human Chromosomes For each human chromosome, indicated on the x-axis, the number of NUMTs (y-axis, on the left) common to human and chimpanzee (white columns) and specific to humans (black columns) are shown. An open circle indicates the chromosome size in millions of base pairs (Mbp; y-axis on the right).

The distribution of human-specific NUMTs in human chromosomes is not proportional either to the chromosome size or to the total number of NUMTs present in the chromosome (Figure 4). In Chromosomes 13 and 20, human-specific NUMTs represent 37% and 50%, respectively, of the NUMT insertions detected in the chromosome. More surprisingly, in Chromosomes 18 and Y there is a proportionally higher number of human-specific NUMTs (2/3 present in each chromosome; see Figure 4), whereas at the genome-wide level about 13% (27/211) of NUMTs are specific to humans. The high number of human-specific NUMTs on the Y chromosome is particularly intriguing since this chromosome is 4-fold less present in the human population than the other chromosomes (it is the only haploid chromosome, present only in males). Additionally, the NUMT value for the Y chromosome may be an underestimate, since its large heterochromatic portion has not yet been sequenced. Since no more NUMTs are available to increase sampling size, we cannot formally distinguish between a founder effect and an increased insertion rate in Chromosomes 18 and Y above that in other chromosomes during recent human evolution.

### NUMTs Mainly Integrate in Known or Predicted Genes

**Figure 5:**
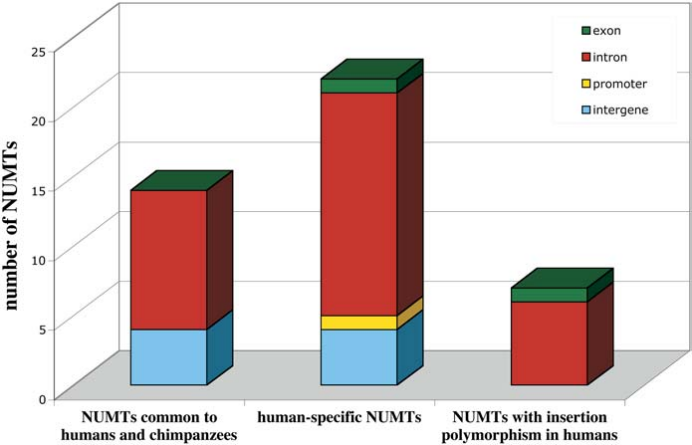
Insertion Sites of NUMTs in the Human Genome Histogram of the insertion sites of NUMTs in the human genome. Only NUMTs tested in human and in chimpanzee samples are shown. This includes the 27 NUMTs specific to humans and absent from chimpanzees (21 present in all individuals tested and 6 with insertion polymorphism in humans), one additional NUMT with insertion polymorphism, previously described, see text, and 14 NUMTs common to human and chimpanzee, out of 183 found by BLAST search. Colors of the blocks indicate the different target sites. For details see Table 3.

**Figure 6:**
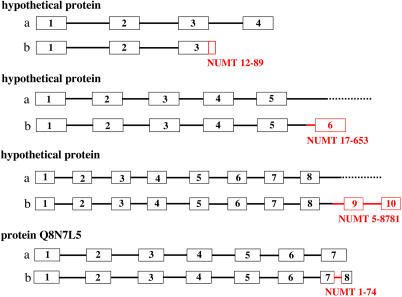
Scheme Representing Some NUMT Insertions in Genes Four known or predicted genes, found in loci with NUMT insertion in humans, have been schematically represented either in the absence (A) or in the presence (B) of the insertion. Boxes represent exons, and thick lines represent introns. Red boxes and lines indicate the sequence corresponding to the NUMT, which has been identified for each case. A dotted line in (A) indicates that, in the absence of insertion, the exon/intron pattern was not identified by gene identification programs. Representation not to scale.

The integration of NUMTs in the human genome takes place, surprisingly, mainly in known or predicted coding or regulatory regions. As indicated in Table 3 and Figure 5, out of 28 human-specific NUMTs, 22 integrate in a known or predicted intron, one in an exon, and one in a promoter region. Only 4/28 NUMTs are in intergenic regions. This is also the case for older NUMTs, common to humans and chimpanzees, where 10/14 NUMTs are inserted in intron regions, and 4/14 in intergenic regions. All seven of the most recent integrations, those displaying insertion polymorphism, were found in exons or introns. In summary, about 80% of NUMTs are inserted in known or predicted introns/exons, which together should cover about 25% of the human genome (Venter et al. 2001). Analysis of the position of NUMTs inside introns reveals that one NUMT, 12-89, was inserted exactly at the splice-donor site of the last predicted intron (Figures 2 and 6). The other 31 NUMTs appear to be randomly integrated within the introns, although in two cases the insertion generates one or two new exons in the predicted proteins (NUMT 17-653 and NUMT 5-8781, respectively); see Figure 6. Moreover, NUMT 1-74, which is the only NUMT found inserted within an exon, splits the last exon of the gene *Q8N7L5* into two, and the NUMT itself becomes a new intron (Figures 2 and 6). Thus, for at least four NUMTs, out of the 33 that are inserted in genes, the exon/intron pattern looked modified after integration of the sequence of mt origin, essentially by a change in the number of exons. We would expect these to be the most likely candidates to perturb gene function.

**Table 3:**
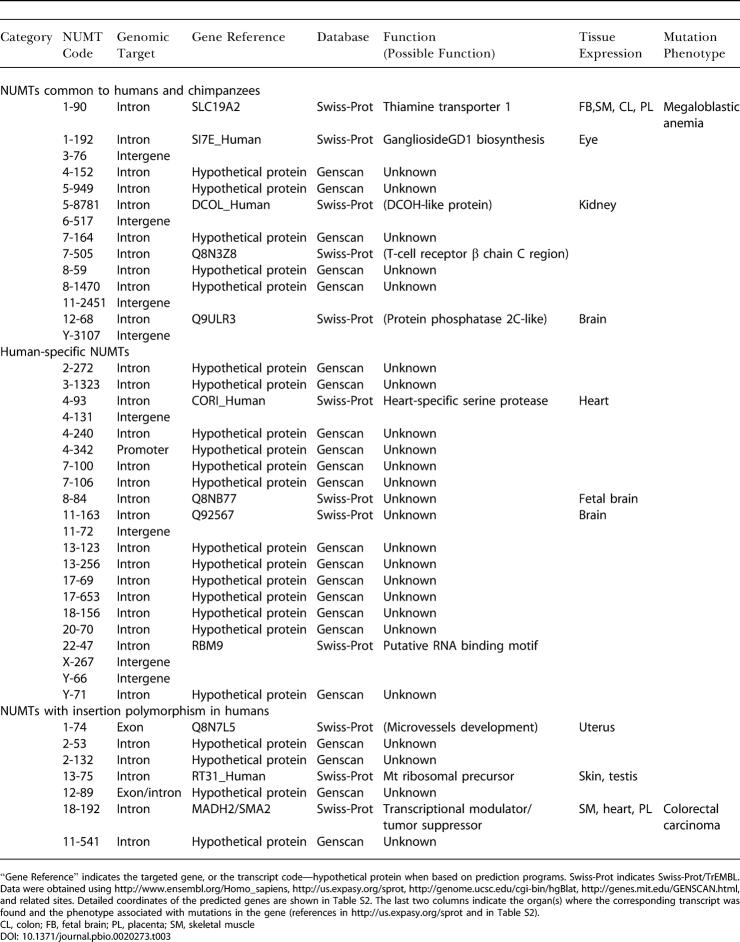
Insertion Sites of NUMTs in the Human Genome

“Gene Reference” indicates the targeted gene, or the transcript code—hypothetical protein when based on prediction programs. Swiss-Prot indicates Swiss-Prot/TrEMBL. Data were obtained using http://www.ensembl.org/Homo_sapiens, http://us.expasy.org/sprot, http://genome.ucsc.edu/cgi-bin/hgBlat, http://genes.mit.edu/GENSCAN.html, and related sites. Detailed coordinates of the predicted genes are shown in Table S2. The last two columns indicate the organ(s) where the corresponding transcript was found and the phenotype associated with mutations in the gene (references in http://us.expasy.org/sprot and in Table S2)

CL, colon; FB, fetal brain; PL, placenta; SM, skeletal muscle

Twenty-one out of 33 NUMTs are inserted in predicted genes, and the other 12 in known genes, including a heart-specific serine protease and a thiamine transporter (Table 3). Interestingly, three of the genes targeted by NUMTs with insertion polymorphism in humans are *MADH2,* a tumor-suppressor gene, mutated in colorectal carcinoma (Eppert et al. 1996; NUMT 18-192); a gene coding for a homologue of the thrombospondin gene (an angiogenesis inhibitor that retards tumor growth; Bogdanov et al. 1999; NUMT 1-74); and a mt ribosomal precursor (NUMT 13-75; Table 3). For NUMT 1-74, which is inserted in an exon, and for NUMTs 18-192 and 13-75, both inserted in an intron, it is not known if individuals carrying the insertion are mutated for these genes.

These findings suggest that in humans, the insertion of NUMTs is elevated in gene-containing regions of the genome. Insertions in such regions are potentially mutagenic. Interestingly, at least two cases of NUMT insertions—one in an exon, the other in an intron region—associated with diseases have been recently reported in humans (Borensztajn et al. 2002; Turner et al. 2003).

## Discussion

Integration of mt genes into the nuclear genome is a physiologically important process that contributes to the origin and evolution of the eukaryotic cell (Margulis 1970), and the transfer of entire genes from mitochondria to the nucleus appears to be continually active in some plants (Knoop et al. 1995). Although the transfer of entire genes seems to have ended in animals, DNA fragments of mitochondrial origin continue to integrate in the nuclear genome. In the present study, we examined the extent and the consequences of this process in humans and in chimpanzees.

### NUMTs Are Present in the Human Genome and Display Insertion Polymorphism

The direct investigation of samples of different individuals provided in this study clearly demonstrates the presence of DNA fragments of mitochondrial origin in the nuclear genome of humans, as previously suggested by the analysis of the databases (Mourier et al. 2001; Woischnik and Moraes 2002) and by a few tests in cells (Tourmen et al. 2002). Although the presence of a single NUMT was previously shown in vivo (Zischler et al. 1995), our study provides direct evidence that the large colonization of the human genome by NUMTs detected in silico, an outcome of the sequencing of the entire human genome, corresponds to the in vivo situation. We can thus exclude that, at least for the tested loci, NUMTs result from contaminations during the sequencing process, a situation that could not be previously ruled out formally (see comments in Venter et al. 2001, and in Mourier et al. 2001).

Our investigation of the distribution of NUMTs in human populations, which includes some of the less divergent among the 211 NUMTs, reveals that in most cases NUMTs are present in all the individuals tested, and therefore these sequences have colonized the nuclear genome of all major human populations. However, six NUMTs described here and one described previously (Zischler et al. 1995; Thomas et al. 1996) are present only in some individuals. These seven NUMTs, not fixed within the human population, must have been recently acquired. Since they are present in individuals within each ethnic group, their insertion most probably occurred after the origin of modern humans and before the emergence of distinct ethnic groups (see also below). We did not detect NUMTs restricted to only one or a few ethnic groups. Furthermore, these seven NUMTs appear to have colonized the genome of human populations at different rates. Indeed, the frequency of alleles carrying the insertion varies greatly according to the NUMT (from 21% to 98% in our samples). This suggests that each sequence exhibits different colonization dynamics, involving the time of insertion and/or the expansion rate of the founder individual(s). Moreover, the distribution of each NUMT is unequal between ethnic groups, and a larger analysis of human populations will be necessary to reveal distinct population patterns and to perform phylogenetic studies. Nevertheless, most individuals tested had a unique combination of these seven NUMTs, suggesting that the individual pattern of NUMT insertion polymorphism can be useful as genetic fingerprints for familial pedigree studies. We expect that other NUMTs displaying such polymorphism will be discovered when larger population samples are examined. The locus 14-1023, which contains a NUMT according to the genome sequence, does not amplify a NUMT-containing fragment in all of 21 individuals. If this does not represent a sequencing artifact, it may be a further example of insertion polymorphism. Furthermore, we propose that the number of NUMT insertion polymorphisms is currently underestimated, since sequencing of the human genome was done only on a limited number of individuals (Lander et al. 2001).

Several independent markers are needed to accurately retrace the phylogeny of human populations (Rosenberg et al. 2002), and insertion polymorphism is particularly interesting because of the low fixation rate and lack of reversion, unlike markers such as single nucleotide polymorphisms. Each of the six insertion polymorphisms described here is a rare event, and if a neutral genetic marker, it provides an important tool for tracing human dispersal.

### Insertion Rate of NUMTs in H. sapiens

An important question concerning the integration of DNA sequences in the nuclear genome is their rate of colonization. For exogenous sequences like NUMTs, this has not been investigated in vivo. To determine the extent of colonization of a given genome, it is necessary to compare the insertions within this genome with those of a closely related species. To date, a comprehensive analysis of the presence of NUMTs has been done for several complete nuclear genomes (Ricchetti et al. 1999; Mourier et al. 2001; Tourmen et al. 2002; Richly and Leister 2004), but the colonization rate of these sequences was not investigated, because of the absence of data in closely related species. In the case of *H. sapiens,* although a proportion of NUMTs present in its genome appears as ancient insertions (see Results; Tourmen et al. 2002; Bensasson et al. 2003), it is not clear how many NUMTs were inserted in primate ancestors and how many specifically colonized the human genome. Chimpanzee *(P. troglodytes),* a species closely related to humans and whose evolutionary relationship with humans has been widely investigated, is an ideal candidate for a comparative analysis. Moreover, the high level of identity (more than 98%) between the two species (Chen et al. 2001; Fujiyama et al. 2002) allows the investigation of the respective genomes using molecular tools. No previous analysis in vivo showed the presence of NUMTs in the chimpanzee genome. By direct PCR amplification and sequencing of chimpanzee samples, we found that out of 41 NUMTs, 14 are also integrated in the chimpanzee genome (see above) and were therefore present in the common ancestor of humans and chimpanzees. However 27 NUMTs are absent from the chimpanzee genome, and are therefore recent acquisitions in *H. sapiens.* For NUMTs fixed in the human genome (not displaying insertion polymorphism), we do not expect this value to increase significantly, since our analysis was made on essentially the entire human genome.

Among all NUMTs detected in the human genome, only about 13% (27 out of 211, or 28 if we include NUMT 11-541) are specific to H. sapiens and have integrated in the human genome in the last 4–6 Myr, after the split of the two species from their common ancestor (Chen and Li 2001). This corresponds to an average of one integration in the germline each 180,000 y, or 5.4 insertions per Myr, a value remarkably close to that estimated by a phylogenetic analysis (5.1 insertions per Myr), which assumed a uniform insertion rate over time (Bensasson et al. 2003). However, the rate of integration of the more recent NUMTs may not be consistent with a constant insertion rate. Indeed, out of 28 human-specific NUMTs, seven display insertion polymorphism and are present in all populations; thus they must have appeared early after the origin of modern humans. The date of this origin is still uncertain. If we assume that NUMTs with insertion polymorphism have been inserted at the same rate as the older NUMTs (fixed in the population), then they must have integrated in the genome of the human ancestor not earlier than 1.4 Myr ago, long before the origin of modern humans, and after the spread of Homo erectus out of Africa (1.7 Myr ago). Living humans would still be polymorphic for these NUMTs, as a result of interbreeding of the nonmodern human populations with modern humans (Templeton 2002). On the contrary, if we assume that these insertions are more recent, as suggested by the poor allelic presence of most of them in present populations, then they must have appeared shortly before the expansion of modern humans, estimated at about 100,000 y ago (Templeton 2002). In this case, their integration rate would be significantly higher than that of NUMTs inserted in the human genome in the previous 4–6 Myr. If the latter is the case, this strikingly high difference in the rate of colonization of the human genome may be due to a founder effect (i.e., the sporadic expansion of individuals carrying specific NUMTs) or, alternatively, to a genuine increase in the integration rate in modern humans. A third possibility is that there is no increase in the insertion rate in modern humans and that the number of “recent” NUMTs is overestimated because they include unfixed NUMTs that are destined to be lost eventually. In this latter case, we would need to assume that at least some of the NUMTs with insertion polymorphism are not neutral and are associated with a selectable phenotype. Although this may not be true for NUMTs 2-132 and 13-75, present in more than 95% of alleles tested, we cannot exclude that the low allelic presence (21%) of NUMTs 1-74 and 12-89 (both inserted in the context of an exon) is the result of the progressive counterselection of a defective phenotype; this would have implications for the mutagenic potential of NUMTs (see below).

Compared to 28 NUMT insertions in the human nuclear genome in the last 4–6 Myr, it has been calculated that about 5,000 new insertion events of Alu repeats have occurred in the human genome in the same timescale (reviewed in Batzer and Deininger 2002). This large difference may be due to the fact that Alu elements are endogenous sequences that can be amplified by reverse transcriptase provided by long interspersed elements and inserted in the genome using L1 endonuclease (Batzer and Deininger 2002), whereas the integration of NUMTs depends only on the availability of DSBs and of the repair machinery (Ricchetti et al. 1999; Yu and Gabriel 1999). This suggests that retrotranscription/integration mechanisms increase the insertion efficiency of DNA sequences by two orders of magnitude. Alternatively, the limited number of NUMTs in the human genome may result from the selection process, if NUMTs preferentially integrate in coding regions (see below).

### Consequences of the Preferential Integration of NUMTs in Genes

Contrary to previous findings, which indicated that NUMTs were inserted mostly outside annotated genes (Woischnik and Moraes 2002), we find that NUMTs preferentially integrate in known or predicted genes. The availability of a more powerful database analysis on genome Web sites and the resulting increase in the number of potential new genes may explain this different evaluation. Unlike previous analyses, we investigated more recent insertions, frequently characterized by short sequences, which may have been missed in earlier studies. Moreover, we find that all of the most recent integrations, namely NUMTs with insertion polymorphisms, are integrated in genes. In cases where it was possible to identify the gene, its transcript was detected in one or more tissues (Table 3). Among the targeted genes we found *MADH2,* a transcriptional modulator with tumor-suppressor properties (Eppert et al. 1996), and a gene involved in microvessel development, and in both cases the NUMTwas present only in some individuals. In these cases it is not known whether the insertion has affected the function of the gene. Recent findings suggest that transcription promotes DNA breaks (Gonzalez-Barrera et al. 2002). Insertion of NUMTs is, at least in yeast, dependent on DSBs (Ricchetti et al. 1999), and in humans it is frequently associated with a hallmark of NHEJ, a DSB repair mechanism (our study). It is therefore possible that highly transcribed genes, perhaps carrying DSBs, are the preferential targets for the insertion of NUMTs.

Only eight out of 41 NUMTs were found in intergenic regions, which should represent 75% of the human genome (Venter et al. 2001). The Genscan program, which detected several insertion targets in our analysis, identifies around 20% more genes than previous estimates (Das et al. 2001), but this does not significantly change the proportion of the genome that is noncoding. Approximately 80% of NUMTs are in coding regions, and we consider this to be a statistically significant event. Interestingly, this was not the case for yeast, where NUMTs integrated with 41-fold preference in intergenic regions (Ricchetti et al. 1999). The intronless structure of the yeast genome may explain this difference, since NUMTs inserted in genes would essentially target exons in yeast and would be selected against if deleterious. In humans, the high content of introns would buffer most of the mutagenic potential of these insertions. Nevertheless, we expect that a fraction of insertions would be harmful also in humans. Although most of the analyzed NUMTs are internal to introns, in at least three cases the insertion modified the exon/intron pattern, and this may be mutagenic. Our analysis indeed confirms the recent finding that two NUMTs*,* occasionally found as new insertions in the human genome, and associated with diseases in humans, are inserted in genes, either in an exon or at the junction between exons and introns (Borensztajn et al. 2002; Turner et al. 2003). Therefore, it is likely that future insertion events in the human genome would also preferentially target genes.

An intriguing finding is that NUMT 12-89 is located exactly at the splice-donor site of the predicted intron. Insertion in a splice-related site was found also in human factor VII gene, where a 251-bp NUMT integrated a splice-acceptor site in a patient with severe plasma factor VII deficiency (Borensztajn et al. 2002). Taken together, these results account for two insertions at intron-splice sites out of 45 NUMT insertion sites analyzed (42 NUMTs in our study and present in human populations and three more NUMTs found in one or more individuals and correlated with a disease; Willett-Brozick et al. 2001; Borensztajn et al. 2002; Turner et al. 2003). The limited sampling size does not permit us to determine if these finding are significant, although it is tempting to speculate that splice sites can be favored targets for the insertion of NUMTs.

Is the rate of de novo insertions in the human germline limited to one each 180,000 y, or even ten times higher? The number of NUMTs detected as very recent insertions in one or a few individuals suggest that the insertion rate of these sequences in humans is currently dramatically underestimated (Willett-Brozick et al. 2001; Borensztajn et al. 2002; Turner et al. 2003). Three new insertions of NUMTs have been found in living individuals, occasionally detected because of the search for the cause of a mutated phenotype. It seems reasonable to predict that a wider search would reveal many more NUMTs present in single or in small groups of individuals. Since NUMTs preferentially target genes, a fraction of these NUMTs could also be connected with diseases. Moreover, one expects that harmful insertions, whose probability increases as genes become preferential targets, would be subject to negative selection and thus removed from the gene pool. Thus the low fixation rate of NUMTs in the human genome may be a direct consequence of their preference for insertion in genes. NUMTs specific to the genome of H. sapiens and widespread in major human populations may represent only a small fraction of insertions that have occurred continually in the human genome. We propose therefore that the insertion of NUMTs, previously considered as functionless (Perna et al. 1996; Hazkani-Covo et al. 2003), at best an evolutionarily important but essentially harmless process, is a potentially mutagenic process, challenging the functional integrity of the human genome.

Remarkably, the integration of NUMTs in the nuclear genome can be accelerated under increased induction of DSBs. In the yeast nuclear genome, where only 34 NUMTs were detected, new NUMTs are integrated at an induced DSB site with a high frequency (10^−3^−10^−4^ per repair event; Ricchetti et al. 1999). In keeping with this notion, a de novo insertion was reported on Chromosome 7 for a patient conceived during the Chernobyl nuclear meltdown (Turner et al. 2003). By analogy to our previous findings in yeast (Ricchetti et al. 1999), it is possible that this novel insertion is the consequence of a de novo DSB in the chromosome resulting from radiation exposure. Consistent with this view, a NUMT has been found inserted at the breakpoint junction of a familial constitutional reciprocal translocation, also associated with the occurrence of a DSB (Willett-Brozick et al. 2001).

The fixation rate and the insertion strategy used by NUMTs are probably the prototype for the integration of exogenous sequences in the nuclear genome. Like NUMTs, sequences lacking specific amplification and integration mechanisms would rely on occasional DSBs to integrate in chromosomes. Coding or transcribed sequences could represent the preferred insertion target for these sequences as well. This strategy is in sharp contrast with the integration procedure of retrotranscribed elements, which have successfully colonized the human genome and only rarely target coding regions (Lander et al. 2001).

In conclusion, we provide direct evidence that NUMTs are present in the human and in the chimpanzee genomes and that the insertion polymorphisms of six NUMTs reveal new markers for the study of human population genetics. Further, our in vivo analysis reveals that on average one new NUMT is fixed in the human genome each 180,000 y, although during the expansion of modern humans the fixation rate of NUMTs may have increased. The frequency of insertion of NUMTs may represent the genuine fixation rate of exogenous sequences colonizing the human nuclear genome. Strikingly, NUMTs preferentially integrate in introns and in exons, and they are thus potentially mutagenic, and novel NUMT integrations have been shown to be associated with diseases in humans. The recent case of de novo insertion of a NUMT following the Chernobyl accident, if not coincidental, provides a compelling example of how environmental insults can drive NUMTs to colonize the nuclear genome and induce genetic dysfunctions in humans.

## Materials and Methods

### 

#### BLAST search

The human mtDNA sequence (Anderson et al. 1981) was compared to the “Homo sapiens genomic contig sequences” database version of April 11, 2003, using the National Center for Biotechnology Information (NCBI, Bethesda, Maryland, United States) “BLAST the Human Genome” server (http://www.ncbi.nlm.nih.gov/genome/seq/page.cgi?F=HsBlast.html&&ORG=Hs). The blastn program was used with default parameters on April 24, 2003. Only output parameters were changed to 1,000 descriptive lines and to 1,000 segment alignments. In a few cases (see Table 1) results of previous searches (July 2001 and January 2003) were also used. BLAST output results were saved locally in a text format and parsed using the readblastn script (see Tekaia et al. 2000), so that results were presented in a table format, including the query sequence, its size, the hit sequence, its size, the blastn E-value, the percent identity, the percent similarity, the matching segment size, and its coordinates on the query sequence as well as on the hit sequence. Only scores less than or equal to 10^−15^ have been selected. Each selected sequence was further aligned with the mtDNA sequence using http://www.ncbi.nlm.nih.gov/blast/b12seq/b12.html.

#### Amplification and sequencing of NUMTs from human and chimpanzee cells

PCR amplification was performed on lysed cells originating from the buccal mucosa of healthy volunteers. Appropriate informed consent was obtained from human subjects. Pygmy samples (two Biakas and two Mbuti pygmies) were obtained as purified DNA from Coriell Institute (Camden, New Jersey, United States). Purified chimpanzee DNA, obtained either from tissues or from fecal material, was a kind gift from J.-P. Vartanian at the Pasteur Institute (Paris, France). For both PCR strategies described in the text, primer sequences are available upon request. Cell lysis was performed by incubating fresh cells overnight at 55 °C in a Tris-EDTA buffer (pH 8.5) in the presence of 200 μg/ml of proteinase K. PCR amplification was performed with 30 cycles of denaturation (1′ at 94 °C), annealing (1′ at 68 °C), and DNA synthesis (3′ at 72 °C) using Invitrogen (Carlsbad, California, United States) *Taq* polymerase. In heterozygous samples, a specific stochiometry of the two bands was found for each couple of primers used. Amplified NUMTs have been sequenced by specialized commercial services, using PCR amplification bands purified by gel extraction.

#### Calculation of the insertion time of NUMTs

The age of insertion of NUMTs was estimated using, as reference, the sequence divergence of the NUMT from the mtDNA. We assumed that the NUMT, when inserted into the nuclear genome was identical to the corresponding mt sequence. We also assumed that, once inserted into the nuclear genome, the NUMT mutated at the same rate as the nuclear genome, μN, which corresponds, for noncoding sequences, to 2.5 × 10^−8^ mutations per nucleotide per generation, or 1.25 × 10^−9^ mutations per nucleotide per year, assuming a generation time of 20 y (Nachman and Crowell 2000). By comparison, the original sequence remaining in DNA is assumed to have undergone mutation at the rate, μM, of 1.7 × 10^−8^ substitutions per nucleotide per year, excluding the D-loop (Ingman et al. 2000). Thus, from the date of insertion (in Myr from the present) the sequence divergence between the NUMT and the cognate mitochondrial sequence is expected to be nearly the sum of mutations accumulated in each compartment (the possibility of compensation by two identical mutations is negligible given the limited divergence). It follows that the date of insertion, *i,* is given by *i* = *d*/(μM + μN), where *d* is the frequency of sequence divergence between the NUMT and present mtDNA sequence. As an example, for a sequence 300 bp long, 94% identity to mtDNA corresponds approximately to an insertion time of 3.3 Myr, and 96% to 2.2 Myr.

## Supporting Information

Table S1 Sequence Analysis of NUMTs in the Human Genome (53 KB DOC).

Table S2 Coordinates of the Genes Where NUMTs Are Inserted in the Human Genome (63 KB DOC).

### Accession Numbers

The NCBI (http://www.ncbi.nlm.nih.gov/genome/seq/page.cgi?F=HsBlast.html&&ORG=Hs) accession number for the human mtDNA sequence is AB055387.

## Abbreviations

DSB: double-strand break
mt: mitochondrial
Myr: million years
NHEJ: non-homologous end-joining
NUMT: nuclear DNA sequence of mitochondrial origin
