# Supplementary material for: Continued Colonization of the Human Genome by Mitochondrial DNA
Source: PLoS Biol. 2004 Sep 7;2(9):e273. doi: 10.1371/journal.pbio.0020273 (PMC515365; doi:10.1371/journal.pbio.0020273)
Supplement: Supplementary file 1 [file pbio.0020273.st001.doc]

1<br>NUMT code	2<br>NCBI identification	3<br>position on the contig	4<br>E-value	5<br>orient	6<br>mt annotation
1-74	ref |NT_004511.14 |Hs1_4668	8190394-8190467	2.0 E-31	Plus	ATPase6
1-90	ref |NT_00668.14 |Hs1_4825	11270701-11270606	1.0E-32	Minus	ATPase8
1-192	ref |NT_034383.1 |Hs1_34545	703878-703686	7.0 E-44	Plus	D-loop
2-53	ref |NT_005367.11 |Hs2_5524	63739-637339	8.0 E-19	Minus	16S rRNA
2-132	ref |NT_037536.1 |Hs2_37540	1114991-1114860	1.0 E-63	Minus	tRNE Phe-12S rRNA
2-272	ref |NT_037528.1 |Hs2_37532 [a]	217411-217140	e-116	Minus	CoI
3-76	ref |NT_022423.13 |Hs3_22579	520262-520337	2.0 E-25	Plus	tRNAs Gln,fMet
3-1323	ref |NT_022475.11 |Hs3_22631	2807840-2806518	0.0	Minus	12S rRNA-16S rRNA
4-93	ref |NT_006238.9 |Hs4_6395	7474025-7473933	2.0 E-35	Minus	CytB
4-131	ref |NT_022853.13 |Hs4_23009	3534207-3534337	2.0 E-53	Plus	12S rRNA
4-152	ref |NT_006316.14 |Hs4_6473	14379712-14379561	2.0 E-56	Minus	16s rRNA
4-240	ref | NT_0022760.12_Hs4_22916	3410282-3410043	1,00E-82	Minus	16srRNA
4-342	ref |NT_016407 |Hs4_16563 [a]	1098580-1098239	e-143	Minus	CoIII
5-949	ref |NT_006576.10 |Hs5_6733	1196410-1195763	3.0 E-80	Plus	CoI
5-8781 up [d]	ref |NT_006721.2 |Hs5_6878 [b]	88846120-90708600	0.0	Plus	CoI>CytB
5-8781 dw [d]	ref |NT_006721.2 |Hs5_6878 [b]	88846120-90708600	0.0	Plus	CoI>CytB
6-527	ref | NT_007299.12_Hs6_7456	105429-105945	0.0	Plus	16srRNA
7-100	ref |NT_007914.11 |Hs7_8071	6228643-6228743	4.0 E-42	Plus	tRNA Val+16S rRNA
7-106	ref |NT_007758.9 |Hs7_7915	6234778-6234883	4.0 E-36	Plus	NADH-5
7-164	ref |NT_007819.12 |Hs7_7976	44589071-44589227	1.0 E-66	Minus	CoI
7-505	ref |NT_007914.10 |Hs7_8071	2935784-2935305	e-110	Minus	tRNA+16srRNA
8-59	ref | NT_008251.12_Hs8_8408	4617119-4617177	3.0 E-15	Plus	12srRNA
8-84	ref |NT_008292 |Hs8_8449	25015145-25015062	6.0 E-35	Minus	CytB
8-1470	ref |NT_023716.2 |Hs8_23872 [b]	308815-310072	0.0	Plus	tRNALys+ATPase8
11-72	ref |NT_033899.4 |Hs11_34054	5495720-5496372	9.0 E-28	Minus	NADH-5+tRNAGlu
11-163	ref |NT_033927.4 |Hs11_34082	3917357-3917515	4.0 E-70	Minus	CoI
11-2451	ref | NT_009307.13_Hs11_9464	1935963-1933514	0.0	Minus	12srRNA-16srRNA
12-68	ref | NT_009711.14_Hs12_9868	1085706-1085773	3.0 E-18	Plus	NADH-1-tRNAIle
12-89	ref | NT_009781.14_Hs12_9938	3483591-3483503	6.0 E-38	Minus	NADH-1
13-75	ref | NT_024524.12_Hs13_24680	9916979-9917049	5.0 E-20	Plus	CoIII
13-123	ref | NT_024524.12_Hs13_24680	25120382-25120260	1.0 E-36	Minus	NADH-2
13-256	ref |NT_009952.10 |Hs13_10109	23135849-23136104	e-135	Plus	12S rRNA
14-1023	ref | NT_026437.10_Hs14_26604	12874363-12873343	0.0	Plus	tRNAAsn-CoI
17-69	ref |NT_010755.12 |Hs17_10912	3763349-3763416	5.0 E-23	Plus	NADH-3
17-653	ref |NT_010783.12 |Hs17_10940	5495720-5496372	0.0	Plus	CoI
18-156	ref |NT_077808.1 |Hs18_77857	2832701-2832547	2.0 E-56	Plus	NADH-5
18-192	ref |NT_033905.4 |Hs18_34060	833525-833334	8.0 E-90	Plus	CoII
20-70	ref | NT_011362.7.12_Hs20_11519	20691968-20692037	5.0 E-17	Plus	NADH-5
22-47	ref |NT_011520.8 |Hs22_11677	15525278-15525324	3.0 E-15	Plus	CoI
X-267	ref |NT_011613.10 |HsX_11770	1175206-1175040	e-118	Minus	12S rRNA
Y-66	ref |NT_011878.7 |HsY_12035	14550-14615	1.0 E-26	Minus	CoI
Y-71	ref |NT_011896.7 |HsY_12053	1678469-1678399	1.0 E-29	Minus	12SRNA
Y-3107	ref |NT_011896.7 |HsY_12053	5703124-5706831	6.0E-35	Plus	12SRNA>NADH-2
11-541 [h]	ref |NT_024160.12 |Hs11_24316	194461-193720	0.0	Plus	D-loop

Supplementary Table 1. Sequence analysis of NUMTs in the humans genome
Column 1 indicates the code name of the NUMT and column 2 the NCBI identification for the sequenced fragment containing the NUMT. Column 3 indicates the position of the NUMT on the chromosomal fragment shown in column 2. Column 4 indicates the blastn E-value. Column 5 shows the orientation of the NUMT, with respect to chromosome orientation and column 6 the mt gene(s) covered by the NUMT (according to Anderson *et al*, 1981) and the sign > separates the first and the last mt genes.
