# Supplementary material for: Continued Colonization of the Human Genome by Mitochondrial DNA
Source: PLoS Biol. 2004 Sep 7;2(9):e273. doi: 10.1371/journal.pbio.0020273 (PMC515365; doi:10.1371/journal.pbio.0020273)
Supplement: Supplementary file 2 [file pbio.0020273.st002.doc]

1	2	3	4	5	6	7	8
NUMT code	SWPR/TrEMBL	Ensembl	Genscan	exon	intron size	insertion coordinates	ref
NUMTs common to human and chimpanzee							
1-90	O60779	ENSG00000117479		2-3	6968	3092-3003	1
1-192	Q9BVH7	ENSG00000117069		2-3	184957	113465-113274	
4-152			NT_006189.34	1-2	22706	19805-19956	
5-949			NT_006576.38	21-22	23416	11862-12810	
5-8781	Q9H0N5	ENSG00000132570		8-9	10864	7499-14710	
7-164			NT_007819.1020	1-2	25793	7569-7760	
7-505	Q8N3Z8			1-2	178776	28270-27766	
8-59			NT_008251.39	1-2	47325	29463-29521	
8-1470			NT_008183.136	3-4	160001	150644-152114	
12-68	Q9ULR3	ENSG00000111110		3-4	50639	14213-14146	
human-specific NUMTs							
2-272			NT_037528	7-8	31031	4994-5266	
3-1323			NT_022475.25	2-3	92393	90084-91407	
4-93	Q9Y5Q5	ENSG00000145244		3-4	23138	14453-14361	
4-240			NT_016354.75	1-2	26529	6585-6825	
4-342			NT_016407.11				
7-100			NT_007914.193	5-6	26491	3638-3543	
7-106			NT_007758.157	4-5	35175	28301-28196	
8-84	Q8NB77	ENSG00000156498		14-15	19884	14068-14152	
11-163	Q92567	ENSG00000054965		1-2	129427	87097-87259	
13-123			NT_024524.919	4-5	199950	120538-120660	
13-256			NT_009952.455	1-2	15780	4216-4472	
17-69			NT_010755.37	1-2	20481	13369-13438	
17-653			NT_010783.152	5-6	26364	26228-26364	
18-156			NT_010859.46	39-40	81238	76858-77014	
20-70			NT_011362.455	6-7	9705	3143-3213	
22-47	O43251	ENSG00000100320		1-2	128843	53176-53130	
Y-71			NT_011896.10	1-2	279515	155251-155231	
NUMTs with insertion polymorphism in humans							
1-74	Q8N7L5	ENSG00000169218		7	1173 (1573)	1174	
2-53			NT_005367.8	4-5	27807	18076-18129	
2-132		ENSG00000175387	NT_037536.10	8-9	6742	4293-4425	
12-89			NT_02419.48	3-4	26123	1-89	
13-75	Q92665	ENSG00000102738		1-2	3951	2622-2693	
18-192	Q15796			6-7	13731	11622-11813	2
11-541[h]			NT_024160.3	8-9	2918	1966-2507	

Supplementary Table 2. Coordinates of the genes where NUMTs are inserted in the human genome.
Column 1 indicates the NUMT code, column 2 the SwissProt/Embl identification number of the transcript and column
Ensembl gene identification number. Columns 4 show the Genescan prediction as NCBI identification fragment, and
5 shows the exon number or, when an intron the two flanking exons, where the NUMT is inserted. Column 6 indicates
size of the intron where the NUMT is inserted; for NUMT 1-74 is indicated the exon size before and, in parenthes
after the insertion. Column 7 shows the position of the NUMT inside this intron (or the exon). Column 8 indicates
reference number describing the effect of mutations in the gene; 1 corresponds to Yan et al, 1999. J. Biol. Chem
14926 and 2 to Eppert *et al*, 1996. Cell 86, 543
